# Diagnosis of Fœtal Monstrosities

**Published:** 1882-12

**Authors:** 


### DIAGNOSIS OF FŒTAL MONSTROSITIES.

That it would be exceedingly valuable, if the obstetricians were able to recognize before the delivery, if they have to do with an akencephalus, hydroencephalus, or other fœtal monstrosities, is clear.

Prof. Calderini (*Ref. Deutsche Mediz. Zeit.*, Aug. 10, 1882) has now demonstrated, from theoretical reasons as well—alteration of the pressure and increase of amnion fluid, in consequence of disturbed circulation and different functions of the kidney—as also on three successive cases of his own, that there always exists in cases of fœtal monstrosities hydramnios. This is plausible, and it would be well if further investigations should be made to settle the question, as many a delivery would be made in a different manner, if the obstetrician knew beforehand that he had to deal with a monstrosity.—*Med. and Surg. Rep.*

